# Study on the relationship of depression, anxiety, lifestyle and eating habits with the severity of reflux esophagitis

**DOI:** 10.1186/s12876-021-01717-5

**Published:** 2021-03-20

**Authors:** Rongxin Wang, Jing Wang, Shuiqing Hu

**Affiliations:** 1 Emergency Department, Xuanwu Hospital, Capital Medical University, Beijing, 100053 China; 2 Department of Gastroenterology, Xuanwu Hospital, Capital Medical University, Beijing, 100053 China

**Keywords:** Reflux esophagitis, Depression, Anxiety, Lifestyle, Eating habits, Severity of reflux esophagitis

## Abstract

**Background:**

The etiology of reflux esophagitis (RE) is multi-factorial. This study analyzed the relationship of depression, anxiety, lifestyle and eating habits with RE and its severity and further explored the impact of anxiety and depression on patients’ symptoms and quality of life.

**Methods:**

From September 2016 to February 2018, a total of 689 subjects at Xuanwu Hospital Capital Medical University participated in this survey. They were divided into the RE group (patients diagnosed with RE on gastroscopy, n = 361) and the control group (healthy individuals without heartburn, regurgitation and other gastrointestinal symptoms, n = 328). The survey included general demographic information, lifestyle habits, eating habits, comorbidities, current medications, the gastroesophageal reflux disease (GERD) questionnaire (GerdQ), the Patient Health Questionnaire-9 depression scale and the General Anxiety Disorder-7 anxiety scale.

**Results:**

The mean age and sex ratio of the two groups were similar. Multivariate logistic regression analysis identified the following factors as related to the onset of RE (*p* < 0.05): low education level; drinking strong tea; preferences for sweets, noodles and acidic foods; sleeping on a low pillow; overeating; a short interval between dinner and sleep; anxiety; depression; constipation; history of hypertension; and use of oral calcium channel blockers. Ordinal logistic regression analysis revealed a positive correlation between sleeping on a low pillow and RE severity (*p* = 0.025). Depression had a positive correlation with the severity of symptoms (r_s_ = 0.375, *p* < 0.001) and patients’ quality of life (r_s_ = 0.306, *p* < 0.001), whereas anxiety showed no such association.

**Conclusions:**

Many lifestyle factors and eating habits were correlated with the onset of RE. Notably, sleeping on a low pillow was positively correlated with RE severity, and depression was positively related to the severity of symptoms and patients’ quality of life.

**Supplementary Information:**

The online version contains supplementary material available at 10.1186/s12876-021-01717-5.

## Background

Reflux esophagitis (RE) is a common type of gastroesophageal reflux disease (GERD). According to a recent report, the incidence rate of RE was 9.0–24.6% among healthy adults undergoing endoscopy [1]. In some patients, RE can evolve into Barrett’s esophagus (BE), and the risk of development of cancer in BE is about 0.5% per year. Moreover, 85% of esophageal adenocarcinomas occur in patients with BE [2]. In most patients, resolution of symptoms and RE can be achieved by inhibiting gastric acid secretion. However, in some RE patients, it becomes difficult to relieve the symptoms, even with the use of proton pump inhibitors (PPIs). The etiology of RE is multi-factorial. Lifestyle factors and eating habits such as smoking, drinking, preferences for high-fat and fried foods, and overeating are closely associated with RE [3–6]. In addition, it has been reported in the recent literature that mental disorders, especially anxiety and depression, are closely related to the onset and prognosis of RE [7], which seriously affects the quality of life of patients. Until now, studies on the relationship between these factors and RE have produced inconsistent results with very few studies focusing on factors related to the severity of RE. In China, there is a lack of large sample studies on the effects of anxiety and depression on the severity of RE symptoms and the quality of life of patients. The present study aimed to analyze the relationship of depression, anxiety, lifestyle factors and eating habits with RE and its severity and to further explore the effects of anxiety and depression on RE symptoms and the quality of life of these patients.

## Methods

### Participants and study design

The study was performed at the Gastroenterology Department of Xuanwu Hospital of Capital Medical University from September 2016 to February 2018. During the study period**,** a total of 689 subjects participated in this survey. They were divided into the RE group (patients diagnosed with RE on gastroscopy, n = 361) and the control group (healthy individuals without heartburn, regurgitation and other gastrointestinal symptoms, n = 328). All study participants completed the questionnaire during a face-to-face interview with a physician. The patients of the case group were enrolled from the outpatient Department of Gastroenterology of, Xuanwu Hospital. The case group included 361 adult patients who underwent gastroscopy and were diagnosed with RE. Once a patient was confirmed to have RE on gastroscopy by specialized endoscopists, the questionnaire survey was completed in the outpatient department of gastroenterology on the same day, and the physician retrieved the patient’s medical history from the medical records. The control group included 328 healthy adults without regurgitation, heartburn or other gastrointestinal symptoms who visited the Xuanwu Hospital for a health check-up. The questionnaire surveys of the control group participants were conducted in the medical examination center. The questionnaire surveys included a structured questionnaire [3–6, 8, 9] (Additional File 1), the GERD questionnaire (GerdQ) [10], the Patient Health Questionnaire (PHQ)-9 depression scale [11] and the General Anxiety Disorder (GAD)-7 anxiety scale [11]. The study was approved by the Ethics Committee of Xuanwu Hospital of Capital Medical University (reference number: 2015–11) and was performed in accordance with the Declaration of Helsinki. All participants gave written informed consent for enrollment in this study.

RE was divided into 4 grades according to the Los Angeles (LA) classification as follows [12]: grade A: one or more mucosal breaks < 5 mm, that did not extend between the top of two mucosal folds; grade B: one or more mucosal breaks > 5 mm long that did not extend between the top of two mucosal folds; grade C: one or more mucosal breaks that was continuous between the top of two or more mucosal folds but involved < 75% of the circumference; and grade D: one or more mucosal breaks that involved at least 75% of the esophageal circumference.

The exclusion criteria were age < 18 years, history of gastrointestinal tumors, gastrectomy, peptic ulcer, severe liver or kidney disorder, respiratory failure, heart failure and pregnancy.

### Data collection

The structured questionnaire [3–6, 8, 9] (Additional File 1) included demographic information, lifestyle and eating habits, comorbidities and current medications. The demographic information included name, gender, age, education level, height, weight, marital status, monthly income, job and domicile [3–6]. Lifestyle habits included smoking, alcohol drinking, strong tea, coffee, constipation and sleeping on a low pillow [3]. Eating habits included preferences for sweets, spicy foods, acidic foods, fruits, noodles, fried and fatty foods; the interval between dinner and sleep; and overeating [3]. Comorbidities included hypertension, ischemic heart disease, diabetes mellitus, hyperlipidemia and asthma [8]. Current medications included low-dose aspirin, clopidogrel, calcium channel blockers, angiotensin receptor blockers, statins, hypoglycemic agents, theophylline and β-receptor blockers [5, 9].

### Definitions

A regular smoker was defined as a person who smoked ≥ 1 cigarette/day, for 6 months continuously or cumulatively, in accordance with World Health Organization (WHO) standards [3]. A drinker was defined as a person with a daily alcohol consumption level of > 25 g for men or > 15 g for women, for 6 months continuously or cumulatively, in accordance with the recommendations of the Chinese Ministry of Health [3]. Overeating was defined as continuing to eat beyond a sensation of fullness until unable to eat any more. The interval between dinner and sleep was said to be short if the duration was less than 2 h. Constipation was defined as a stool frequency of less than 3 times a week, dry and hard stools, and difficulty defecating.

Preference was defined as engaging in the habit > 3 days/week, continuously or cumulatively for 6 months [3]. In this study, preferences were recorded for strong tea (> 3 g of tea), spicy foods (e.g., onion, garlic, chili, pepper), fried foods and fatty foods (e.g., fatty, sesame, butter, animal offal), acidic foods (e.g., acid seasoning, yogurt, acidic drinks), sweets (e.g., cream, cake, chocolate) and fruits (e.g., hawthorn, lemon, orange). Pillow height was also recorded, and a low pillow was defined by a height less than 10 cm.

### Questionnaires

The GerdQ questionnaire consists of six items, mainly based on the common symptoms experienced by GERD patients and the impact of the disease on daily life. This questionnaire has six parts: regurgitation, heartburn, upper abdominal pain, nausea, sleep disturbance and the need for additional medication to relieve symptoms. Patients were required to recall the previous 7 days when completing questionnaire, and the scores were added. The sum was the GerdQ score for the patient, and the total score ranged from 0 to 18 points [10]. A higher score reflects more severe symptoms.

The PHQ-9 depression scale is a depression screening scale based on the frequency of depression symptoms in the previous 2 weeks. This scale has nine main parts: little interest or pleasure in doing things; feeling down or hopeless; trouble falling or staying asleep; feeling tired or having little energy; poor appetite or overeating; feeling bad about yourself; moving or speaking slowly or restless; trouble concentrating on things; and thoughts of death. The sum of the points was the patient’s scale score, the total score ranged from 0 to 27 points. The results were classified as follows: 0–4 points indicated normal; 5–9 points was considered mild depression; 10–14 points reflected moderate depression; and 15–27 points represented severe depression [11].

The GAD-7 Anxiety Scale is an anxiety screening scale that mainly asks patients to recall the frequency of anxiety symptoms in their lives in the previous 2 weeks. It included seven main parts: feeling nervous, anxious or on edge; not being able to stop or control worrying; worrying too much about different things; trouble relaxing; being so restless that is hard to sit still; becoming easily annoyed or irritable; and feeling afraid as if something awful might happen. The total sum of points was the patient’s scale score, and the total score ranged from 0 to 21 points. The results were classified as follows: 0–4 points was considered normal; 5–9 points indicated mild anxiety; 10–14 points reflected moderate anxiety; and 15–21 points represented severe anxiety [11].

### Statistical analysis

Continuous normally distributed variables were expressed as mean ± standard deviation (SD) and compared using Student’s *t*-test. Non-normally distributed variables were expressed as median (upper and lower quartile) and compared by Mann–Whitney *U* test. The chi-square test was used to compare categorical data. Multivariate analysis used logistic regression analysis, and odds ratios (ORs) and 95% confidence intervals (CIs) were calculated by logistic regression. Rank correlation analysis used ordinal logistic regression analysis and Spearman rank correlation analysis. All analyses were two-tailed, and a *p* value < 0.05 was considered to be statistically significant. All statistical analyses were performed using SPSS version 22.0.

## Results

### Indications for gastroscopy in the RE group

The indications for gastroscopy among the RE patients mainly depended on the main symptoms and the duration of their history. Among the 361 patients in the RE group, symptoms of heartburn and/or regurgitation were the main indications. The numbers of patients with specific symptoms were as follows: 46 (12.7%) had heartburn only, 71 (19.7%) had regurgitation only, 186 (51.5%) had both regurgitation and heartburn, and 58 (16.1%) had other atypical symptoms. The duration of symptom history ranged from 3 to 6 months in 50.1% cases (Table 1).

**Table 1:** Indications for gastroscopy among patients in the RE group

Main symptoms	n(%*)	Duration of symptoms			
		< 1 month n (%**)	1–3 months n (%**)	3–6 months n (%**)	> 6 months n (%**)
Heartburn (only)	46 (12.7)	2 (4.3)	4 (8.7)	25 (54.3)	15 (32.6)
Regurgitation (only)	71 (19.7)	5 (7.0)	5 (7.0)	36 (50.7)	25 (35.2)
Heartburn and regurgitation	186 (51.5)	2 (1.1)	10 (5.4)	102 (54.8)	72 (38.7)
Pharyngeal paresthesia	19 (5.3)	5 (26.3)	10 (52.6)	4 (21.1)	0 (0)
Dysphagia	12 (3.3)	6 (50.0)	5 (41.7)	1 (8.3)	0 (0)
Dry cough	11 (3.0)	0 (0)	2 (18.2)	6 (54.5)	3 (27.3)
Nausea	6 (1.7)	0 (0)	1 (16.7)	3 (50.0)	2 (33.3)
Upper abdominal pain	10 (2.8)	1 (10.0)	5 (50.0)	4 (40.0)	0 (0)
Total	361	21 (5.8)	42 (11.6)	181 (50.1)	117 (32.4)

^*^%, number of patients with symptom/total number in RE group

^**^%, numbers of patients with that duration of symptom/number of patients with that symptom

### Comparison of demographic data

A total of 361 patients with RE were included in this study. These included 164 males and 197 females for a male:female ratio of 1:1.2. The mean patient age was 49.5 ± 12.9 years (range, 20–82 years). There were 328 healthy individuals in the control group, including 141 males and 187 females for a male:female ratio of 1:1.3. The mean age of the control individuals was 48.9 ± 12.7 years (range, 28–76 years). The sex ratio (*p* = 0.519) and the mean age (*p* = 0.478) did not differ significantly between the two groups.

### Univariate analysis of factors associated with RE

Univariate analysis identified the following factors as associated with RE: low education level; smoking; drinking strong tea; overeating; a short interval between dinner and sleep; preferences for sweets, acidic foods, fruits, noodles, fried and fatty foods; sleeping on a low pillow; constipation; overweight/obesity; history of hypertension; and use of oral calcium channel blockers (Table 2).

**Table 2:** Univariate analysis of factors associated with RE

Risk factors	Number of cases (%)		*p* value*
	RE group (n = 361)	Control group (n = 328)	
*Education level*			
Up to junior high school	95 (26.3)	25 (7.6)	< 0.001**
Higher than junior high school	266 (73.7)	303 (92.4)	
*Marital status*			
Single	17 (4.7)	19 (5.8)	0.524
Married	324 (89.8)	293 (89.3)	0.857
Divorced/Widowed	20 (5.5)	16 (4.9)	0.697
Job			
Unemployed	20 (5.5)	16 (4.9)	0.697
Self-employed	32 (8.9)	22 (6.7)	0.293
Employed	309 (85.6)	290 (88.4)	0.273
*Domicile*			
Rural	41 (11.4)	28 (8.5)	0.218
Urban	320 (88.6)	300 (91.5)	
*Monthly income*			
< $800	55 (15.2)	42 (12.8)	0.360
$800–1500	199 (55.1)	196 (59.8)	0.220
> $1500	107 (29.6)	90 (27.4)	0.523
Smoking	84 (23.3)	50 (15.2)	0.008**
Alcohol drinking	75 (20.8)	64 (19.5)	0.170
Drinking strong tea	87 (24.1)	35 (10.7)	< 0.001**
Drinking coffee	48 (13.3)	60 (18.3)	0.072
Preference for sweets	172 (47.6)	86 (26.2)	< 0.001**
Overeating	200 (55.4)	55 (16.8)	< 0.001**
Short interval between dinner and sleep	192 (53.2)	72 (22.0)	< 0.001**
Preference for spicy foods	128 (35.5)	120 (36.6)	0.758
Preference for acidic foods	74 (20.5)	20 (6.1)	< 0.001**
Preference for noodles	193 (53.5)	88 (26.8)	< 0.001**
Preference for fried and fatty foods	93 (25.8)	59 (18.0)	0.014**
Preference for fruits	93 (25.8)	59 (18.0)	0.014**
Constipation	76 (21.1)	28 (8.5)	< 0.001**
Sleeping on a low pillow	159 (44.0)	49 (14.9)	< 0.001**
*BMI (kg/m*^*2*^*)*			
Normal weight (18.5–23.9)	196 (54.3)	231 (70.4)	< 0.001**
Overweight (24–27.9)	115 (31.9)	76 (23.2)	0.011**
Obese (≥ 28)	50 (13.9)	21 (6.4)	0.001**
*Comorbidities*			
Hypertension	104 (28.8)	42 (12.8)	< 0.001**
Ischemic heart disease	29 (8.0)	15 (4.6)	0.064
Diabetes mellitus	34 (9.4)	20 (6.1)	0.106
Hyperlipidemia	47 (13.0)	30 (9.1)	0.107
Asthma	15 (4.2)	6 (1.8)	0.076
*Current medications (oral)*			
Calcium channel blockers	58 (16.1)	19 (5.8)	< 0.001**
Low-dose aspirin	33 (9.1)	18 (5.5)	0. 068
Clopidogrel	18 (5.0)	9 (2.7)	0.130
Statins	45 (12.5)	29 (8.8)	0.125
Angiotensin receptor blockers	43 (11.9)	25 (7.6)	0. 060
Hypoglycemic agents	32 (8.9)	18 (5.5)	0.088
Theophylline	10 (2.8)	4 (1.2)	0.150
β-receptor blockers	46 (12.7)	30 (9.1)	0.626

*BMI* body mass index. *p-values were derived from Chi-square test. **Significant at the level of *p* < 0.05

### Comparison of anxiety and depression between the two groups

The median GAD-7 anxiety score of the RE group [2.00 (0.00, 7.00)] was significantly higher than that of the control group [1.00 (0.00, 3.00)] (*p* < 0.01). The median PHQ-9 depression score of the RE group [4.00 (1.00, 8.00)] was also significantly higher than that of the control group [1.00 (0.00, 4.00)] (*p* < 0.01). Given that a total score of 0–4 points was considered normal, in terms of composition ratio, the proportions of patients with anxiety and depression were significantly higher in the RE group than in the control group (*p* < 0.01, Table 3).

**Table 3:** Comparison of anxiety and depression in RE and control groups [n (%)]

Groups	PHQ -9 depression score		GAD-7 anxiety score	
	0–4 points	> 4 points	0–4 points	> 4 points
RE group (n = 361)	212 (58.7)	149 (41.3)	235 (65.1)	126 (34.9)
Control group (n = 328)	273 (83.2)	55 (16.8)	281 (85.7)	47 (14.3)
*χ*^2^ value	49.519		38.684	
*p* value*	< 0.001**		< 0.001**	

*p-values were derived from Chi-square test. **Significant at the level of *p* < 0.05

### Multivariate analysis of factors associated with RE

Multivariate logistic regression analysis identified the following factors as significantly associated with RE (*p* < 0.05): low education level; drinking strong tea; preferences for sweets, acidic foods and noodles; sleeping on a low pillow; overeating; a short interval between dinner and sleep; anxiety; depression; constipation; a history of hypertension; and use of oral calcium channel blockers (Table 4).

**Table 4:** Multivariate analysis of factors associated with RE

Factors	*p* value*	OR	95% CI	
			Lower limit	Upper limit
Smoking	0.867	0.946	0.497	1.803
Drinking strong tea	0.004**	2.676	1.362	5.255
Preference for sweets	0.004**	2.049	1.252	3.355
Overeating	< 0.001**	3.446	2.018	5.885
Short interval between dinner and sleep	0.003**	2.163	1.298	3.606
Preference for acidic foods	0.002**	3.593	1.611	8.013
Preference for noodles	< 0.001**	3.259	1.959	5.423
Preference for fried and fatty foods	0.864	0.947	0.507	1.767
Preference for fruits	0.810	1.076	0.593	1.952
Sleeping on a low pillow	< 0.001**	4.343	2.433	7.750
Constipation	0.002**	3.013	1.506	6.030
Overweight /obesity	0.188	1.443	0.836	2.490
Depression	0.032**	2.004	1.126	3.828
Anxiety	0.043**	1.908	1.118	3.669
Low education level	< 0.001**	22.076	12.752	37.267
Hypertension	0.001**	3.474	1.618	7.461
Calcium channel blockers	0.044**	2.722	1.598	7.546

*OR* odds ratio, *CI* confidence interval. *p-values were derived from multivariate logistic regression analysis. **Significant at the level of *p* < 0.05

### Analysis of factors associated with the severity of RE

To further explore whether the factors identified as significantly related to the occurrence of RE also are associated with the severity of RE based on the LA classification, ordinal logistic regression analysis was performed. The results showed that patients sleeping on a low pillow had RE of a higher severity (*p* = 0.025). In the RE group, the numbers of patients according to the LA classification were as follows: 58 (16.1%) LA-A, 281 (77.8%) LA-B, 17 (4.7%) LA-C, and 5 (1.4%) LA-D. The corresponding numbers of patients who reported sleeping on a low pillow were: 18 (31.0%), 129 (45.9%), 9 (52.9%), and 3 (60.0%), respectively. On the other hand, drinking strong tea (*p* = 0.347), a preference for sweets (*p* = 0.226), overeating (*p* = 0.860), a short interval between dinner and sleep (*p* = 0.114), a preference for acidic foods (*p* = 0.636), a preference for noodles (*p* = 0.346), anxiety (*p* = 0.140), depression (*p* = 0.144), low education level (*p* = 0.362), constipation (*p* = 0.839), a history of hypertension (*p* = 0.772), and use of oral calcium channel blockers (*p* = 0.803) were not related to the severity of RE.

### Influence of anxiety and depression on patients’ regurgitation and heartburn symptoms

Among the 361 RE patients, 303 patients had typical symptoms of regurgitation and/or heartburn (typical symptom group), whereas 58 patients had no typical symptoms (atypical symptom group). The median depression and anxiety scores of the typical symptom group were 4.00 (2.00, 8.00) and 4.00 (1.00, 7.00), respectively, and these scores in the atypical symptom group were 3.00 (0.75, 4.25) points and 2.00 (0.00, 6.25) points, respectively. The differences in these scores between the two groups were statistically significant (*p* < 0.05).

**Fig. 1:**
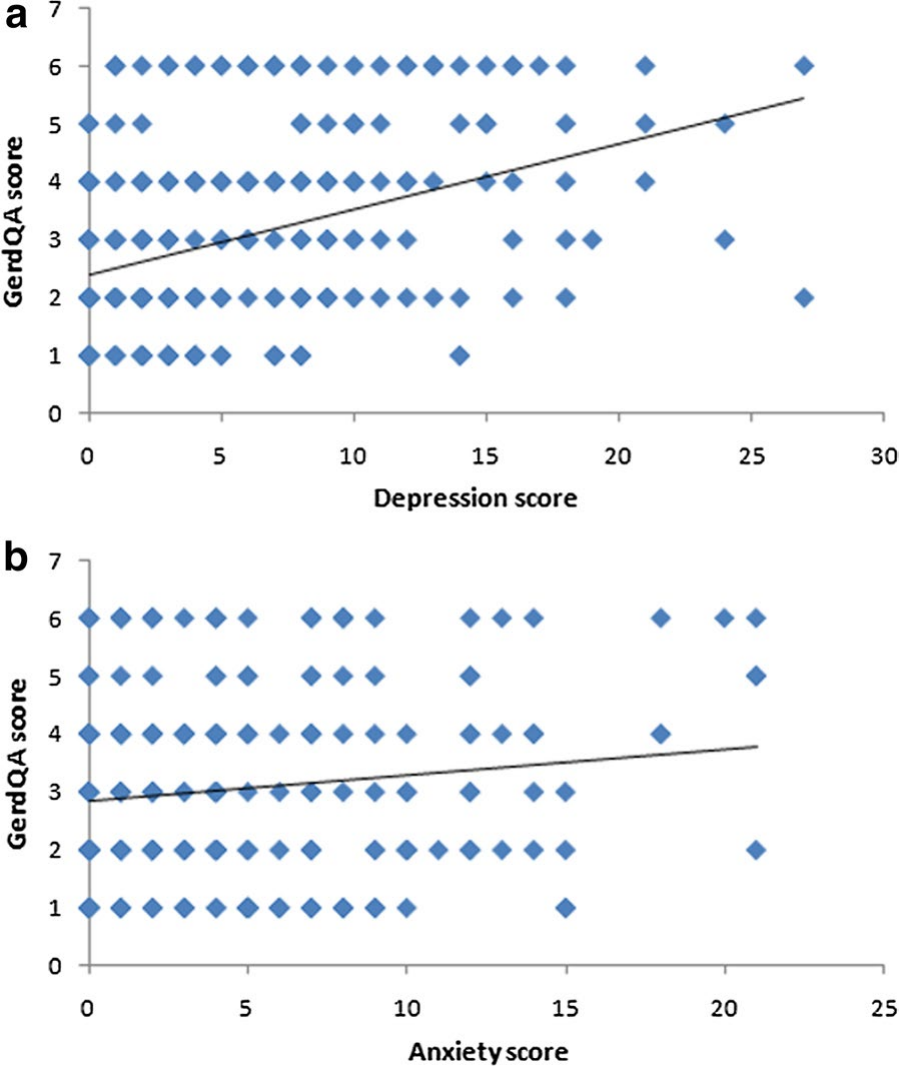
Spearman rank correlation analysis of the influence of **a** depression on patients’ RE symptoms and **b** anxiety on patients’ RE symptoms

According to the GerdQ A score (frequency of regurgitation and heartburn within 1 week), the numbers of patients with scores of 1–6 points were 55, 85, 50, 61, 17 and 35, respectively. Spearman rank correlation analysis showed that the depression score was positively correlated with the severity of RE symptoms (r_s_ = 0.375, *p* < 0.001), while no obvious correlation was found between the anxiety score and the severity of RE symptoms (r_s_ = 0.073, *p* = 0.205) (Figure 1a, b).

### Relationship of anxiety and depression with the impact of RE on patients’ quality of life

The GerdQ C item mainly estimates the impact of GERD on quality of life and has two parts, namely, nocturnal reflux affecting sleep and need for additional medication to relieve symptoms to maintain a normal life in addition to regular treatment. A total of 180 patients were affected in their daily life by GERD (affected group), while 181 patients had no effect (unaffected group). The median depression score [3.00 (1.00, 6.50) vs. 4.00 (2.00, 9.00), *p* = 0.012] and the median anxiety score [2.00 (0.00, 6.00) vs. 3.00 (2.00, 7.00), *p* = 0.043] of the unaffected group were significantly lower than those of the affected group.

**Fig. 2:**
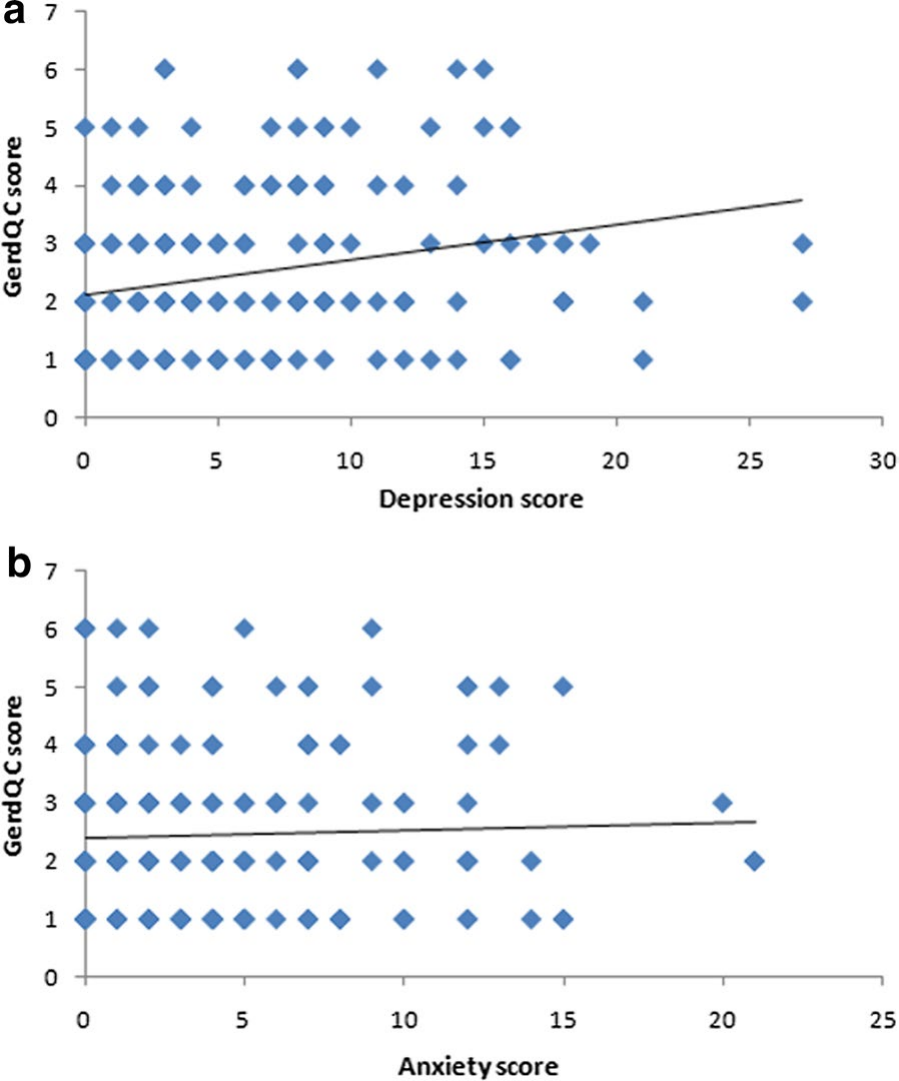
Analysis of the relationship of **a** depression and **b** anxiety with the impact of RE on patients’ quality of life

According to the GerdQ C score, the numbers of patients with scores from 1 to 6 points were 56, 51, 34, 19, 13 and 7, respectively. According to Spearman rank correlation analysis, the severity of depression was positively correlated with the severity of the impact of RE on daily life (r_s_ = 0.306, *p* < 0.001), while the severity of anxiety showed no obvious correlation with severity of the impact of RE on daily life (r_s_ = 0.049, *p* = 0.515) (Fig. 2a, b).

## Discussion

The results of the present study identified low education level, drinking strong tea, overeating, a short interval between dinner and sleep, sleeping on a low pillow, anxiety, depression, constipation, a history of hypertension, use of oral calcium channel blockers, and preferences for sweets, noodles and/or acidic foods as positively related to the onset of RE.

From the perspective of education level, previous studies have reported that a lower education level is a high risk factor for RE, which is supported by the results of this study [4–6]. It is believed that a lower education level may lead to RE by affecting patients’ eating habits, psychological pressure and hygiene habits [6]. A previous study showed that a low income level and living in a rural area were related to the occurrence of RE [5]. However, in the current study, we found no such associations. This might be due to differences in the geographic location and socio-demographic status of the study populations as well as sample sizes.

Tea is considered a healthy drink by two-thirds of people in the world [13]. Studies have shown that drinking tea is one of the factors related to RE [1, 5, 14–18]. Theophylline, the main ingredient in tea, is currently believed to promote gastric acid secretion, relax the lower esophageal sphincter (LES) and reduce LES pressure, thereby increasing the reflux of stomach acid into the esophagus [14]. In a Chinese study, drinking strong tea was closely related to RE [15]. In this study also, we found that the habit of drinking strong tea was positively correlated with RE, but was not associated with its severity. In view of the popularity of tea drinking around the world, tea may be playing an important role in the increasing number of new RE cases. However, further studies are required to determine the quantity, type and quality of tea contributing in the occurrence of RE.

Current opinions on the relationships between fatty foods, spicy foods, sweets, noodles, and fruits and the onset of RE are inconsistent, with some studies showing such associations [14, 15, 18, 19] while others could not confirm such associations [1, 3, 5, 16, 17, 20]. Our study found that preferences for sweets, noodles and acidic foods showed positive correlations with RE, while preferences for fried and fatty foods and fruits were not related to the onset of RE. The differences in these results may be related to the race, geographic location, and eating habits of the study populations.

From the lifestyle perspective, our study showed that constipation, overeating, and a short interval between dinner and sleep were positively associated with the onset of RE. These lifestyle habits can increase abdominal pressure, promote gastric dilatation and increase transient LES relaxation, thereby increasing the probability of gastroesophageal reflux. Previous studies have also reported similar findings [3, 18, 20, 21].

Until now, there were few studies on the factors related to the severity of RE. This study found that sleeping on a low pillow not only showed a positive correlation with the development of RE, but also was closely related to the severity of RE. In patients with a lax LES, sleeping on a low pillow increases the risk of reflux. Moreover, salivary secretion is greatly reducing during night-time sleep, secondary esophageal peristalsis is rare, and esophageal acid clearance at night is significantly delayed. All these factors make reflux during sleep more harmful. Hence, all these reports should be taken seriously [22].

Regarding comorbidities and medications, our study found that a history of hypertension and use of oral calcium channel blockers were closely related to the occurrence of RE but were not associated with the severity of RE. The relationship between hypertension and the onset of RE was considered related to the use of anti-hypertensive drugs, especially calcium channel blockers. Calcium channel blockers can decrease LES pressure, impair esophageal clearance and reduce the amplitude of esophageal contractions, causing reflux [23].

The relationship between body mass index (BMI) and RE has remained controversial [3–6, 24]. In the present study, univariate analysis showed that high BMI and smoking were associated with RE, but these correlations were not found to be significant on multivariate analysis. This may be due to the fact that multivariate regression analysis excluded the interaction between multiple factors. Inconsistent findings have also been reported regarding the link between smoking and RE, with some studies identifying smoking as an independent predictor of RE development [3, 5, 14–16, 21] and others showing no correlation [4, 6, 18, 25, 26]. This might be due to differences in the socio-demographic status of study participants, sample sizes, and data collection and evaluation methods, and further studies are warranted.

In recent years, it had been reported that anxiety and depression are closely related to the onset of RE [7, 8, 21, 27, 28], but there have been few reports on the relationship between these conditions and the severity of RE. In the present study, multivariate analysis confirmed that these mental disorders were associated with the onset of RE, but no correlation was found between the severity of anxiety/depression and the severity of RE. Similarly, these disorders were found to be closely related to the symptoms of RE [7, 8, 27–31]. The pathophysiological mechanisms underlying the relationship between psychological factors and RE symptoms are unclear. Currently, there are several potential explanations. One possibility is that depression and anxiety are secondary to reflux, which in turn increases the sensitivity of patients to reflux symptoms. The second possibility is that delayed gastric emptying in patients with depression and/or anxiety will increase transient LES relaxation due to the influence of the central nervous system, which in turn can aggravate gastroesophageal reflux [8, 27].

Until now, there have been few studies on the influence of depression and anxiety on the severity of symptoms and the impact of RE on the daily life of patients. In the present study, we found that depression was positively correlated with these two aspects, while anxiety had no obvious correlation with them. Recent studies had also showed that after the addition of antidepressants, RE symptoms, nocturnal reflux and the number of additional medications were significantly improved among RE patients, which further confirms this finding [32, 33]. The underlying mechanism of this association is unclear. In the future, we intend to conduct a larger study to validate the findings of this study and further explore the mechanism of this relationship from different aspects of the pathogenesis of anxiety and depression, such as serotonin concentration, which is closely related to esophageal motility [34, 35]. Another point that cannot be ignored is the limitations in the selection of the scales. Unlike the PHQ-9 Depression Scale, the GAD-7 Anxiety Scale lacks judgment of patients’ appetite and sleep, which may affect the results. Therefore, the choice of scale should be further strengthened in the future to further clarify the role of anxiety and depression in the symptoms and daily activities of patients with RE.

Some additional limitations of this study must be noted. First, this was a single-center study and could not be generalized to the other population. Second, this study only included patients with RE and did not consider patients with non-erosive reflux disease. Third, recall bias may have affected the study results, because the questionnaire required the participants to recall events in previous weeks.

## Conclusions

We found that low education level, drinking strong tea, sleeping on a low pillow, overeating, a short interval between dinner and sleep, anxiety, depression, constipation, a history of hypertension, use of oral calcium channel blockers, and preferences for sweets, noodles and acidic foods were closely related to the onset of RE. Sleeping on a low pillow also was positively correlated with the severity of RE. Depression was also positively correlated with the severity of symptoms and the impact of the disease on daily life, while anxiety showed no such associations.

## Supplementary Information

**Additional file 1.** Questionnaire covering factors associated with reflux esophagitis: demographic information, lifestyle habits, eating habits, comorbidities and current medications.

## Data Availability

Upon reasonable request, data from this study are available from the corresponding author.

## Abbreviations

RE: Reflux esophagitis
BMI: Body mass index
CI: Confidence interval
GERD: Gastro-esophageal reflux disease
OR: Odds ratio
LESP: Lower esophageal sphincter pressure
WHO: World Health Organization
BE: Barrett’s esophagus
PPI: Proton pump inhibitor
LA: Los Angeles
PHQ: Patient Health Questionnaire
GerdQ: GERD questionnaire
GAD: General anxiety disorder
