# Supplementary material for: Study on the relationship of depression, anxiety, lifestyle and eating habits with the severity of reflux esophagitis
Source: BMC Gastroenterol. 2021 Mar 20;21:127. doi: 10.1186/s12876-021-01717-5 (PMC7980552; doi:10.1186/s12876-021-01717-5)
Supplement: Supplementary file 1 — Additional file 1. Questionnaire covering factors associated with reflux esophagitis: demographic information, lifestyle habits, eating habits, comorbidities and current medications. [file 12876_2021_1717_MOESM1_ESM.doc]

**Additional file 1: Study on the relationship of depression, anxiety, lifestyle and eating habits with the severity of reflux esophagitis**

**Rongxin Wang^*^**, Jing Wang and Shuiqing Hu

^*^Correspondence: wangrongxin@sina.com

**The Questionnaire**

*Study on the relationship of depression, anxiety, lifestyle and eating habits with the severity of reflux esophagitis*

**Section A- Demographic information**

**Name Gender Age** years

**Weight** kg **Height** cm

**Education level**

□Up to junior high school

□Higher than junior high school

**Marital status**

□Single

□Married

□Divorced/widowed

**Job**

| □Unemployed |
| --- |
| □Self-employed |
| □Employed |
|  |
| **Domicile** |
| □Rural |
| □Urban |
|  |
| **Monthly income** |
| □<$800 |
| □$800-1500 |
| □>$1500 |
|  |

**Section B - Lifestyle and eating habits**

| **Lifestyle habits** | **Yes** | **No** |
| --- | --- | --- |
| Smoking | □ | □ |
| Alcohol drinking | □ | □ |
| Preference for drinking strong tea | □ | □ |
| Preference for drinking coffee | □ | □ |
| Constipation | □ | □ |
| Sleeping on low pillow  (pillow height <10 cm) | □ | □ |

**Eating habits Yes No**

| Preference for sweets | □ | □ |
| --- | --- | --- |
| Overeating | □ | □ |
| Short interval between dinner and sleep (<2 hours) | □ | □ |
| Preference for spicy foods | □ | □ |
| Preference for acidic foods | □ | □ |
| Preference for noodles | □ | □ |
| Preference for fried and fatty foods | □ | □ |
| Preference for fruits | □ | □ |

**Section C -Comorbidities and current medications**

| **Comorbidities** | **Yes** | **No** |
| --- | --- | --- |
| Hypertension | □ | □ |
| Ischemic heart disease | □ | □ |
| Diabetes mellitus | □ | □ |
| Hyperlipidemia | □ | □ |
| Asthma | □ | □ |

**Current medications Yes No**

| Calcium channel blockers | □ | □ |
| --- | --- | --- |
| Low-dose aspirin | □ | □ |
| Clopidogrel | □ | □ |
| Statins | □ | □ |
| Angiotensin receptor blockers | □ | □ |
| Hypoglycemic agents | □ | □ |
| Theophylline | □ | □ |
| β-receptor blockers | □ | □ |

Date
